# Effect of manual osteopathic techniques on the autonomic nervous system, respiratory system function and head-cervical-shoulder complex—a systematic review

**DOI:** 10.3389/fmed.2024.1358529

**Published:** 2024-04-10

**Authors:** Jakub Stępnik, Dariusz Czaprowski, Agnieszka Kędra

**Affiliations:** ^1^Still Academy of Osteopathy, Warsaw, Poland; ^2^SomaticMed Wołomin, Wołomin, Poland; ^3^Department of Physiotherapy, School of Public Health, University of Warmia and Mazury, Olsztyn, Poland; ^4^Center of Body Posture, Olsztyn, Poland; ^5^Faculty of Physical Education and Health, Jozef Pilsudski University of Physical Education in Warsaw, Biała Podlaska, Poland

**Keywords:** osteopathy, autonomic nervous system, heart rate variability, CV4, OMT

## Abstract

**Background:**

Osteopathic manual techniques are now widely used in medicine worldwide. At present, there are no clear conclusions regarding the possibility of affecting the function of the autonomic nervous system (ANS), respiratory system and head-cervical-shoulder complex by manual osteopathic techniques.

**Objectives:**

The aim of the study was to review the current literature regarding the possible impact of osteopathic manual techniques on the state of the autonomic nervous system, spirometric parameters of the respiratory system and the state of the head-collar-shoulder complex.

**Methods:**

Publications have been searched in the following databases: PubMed, Virtual Health Library and Cochrane Central Register of Controlled Trials. The search strategy included keywords related to manual osteopathic treatment, autonomic nervous system, spirometry, respiratory function and head, neck and shoulder pain. The methodological quality of the included studies was assessed. The PRISMA guidelines were used for the systematic review. Studies from 2010 to 2023 were selected.

**Results:**

Using the proposed descriptions and manual searches from the literature of other works, 40 studies were found, out of which 22 were rejected because they did not meet the inclusion criteria. The analysis included: 15 randomized controlled trials, 3 pilot studies.

**Conclusion:**

Studies clearly show the effect of OMT on both spirometric parameters and the condition of the head-collar-shoulder complex. Most often this translates into improved ANS performance, but there are exceptions.

**Systematic review registration:**

https://www.crd.york.ac.uk/prospero/, CRD42023476963.

## 1 Introduction

Osteopathy is a field of medicine (1) that emphasizes a manual procedures approach to healing. These procedures target not only the musculoskeletal and visceral systems but also the nervous system, with a particular focus on the autonomic nervous system (ANS^1^) (2). Researchers have delved into the effects of osteopathic techniques on neural ganglia and the surrounding tissues. For example, manual interventions are designed to counteract tissue densification and cross-linking, or to reverse the consistency changes from sol to gel, which can significantly impair neural function and influence the innervation of various bodily regions (3).

In the realm of the head-cervical-shoulder complex,^2^ studies have predominantly investigated the efficacy of high-velocity low-amplitude (HVLA^3^) manipulations, soft tissue techniques, and gentle mobilizations. These aim to address issues within the cervical ganglia—upper, middle, and lower (stellar)—as well as the thoracic sympathetic ganglia and various cranial ganglia, including the submandibular, palatine, trigeminal, and ciliary (4). Franke et al. highlighted the positive outcomes of these osteopathic manipulations, reporting enhancements in pain management and the mobility of the cervical spine (4). It is important to note that improvements in ANS function due to these techniques can lead to better regulation and recovery of bodily functions, which may have a cascading effect on the overall health of patients with pulmonary or orthopedic conditions. The thoracic spine and lungs also hold clinical significance in osteopathic practice. Techniques like rib rising and thoracic lymphatic pumps have been closely studied for their role in optimizing respiratory functions. This is especially pertinent in the thoracic region of the spine and the rib-transverse joints, where sympathetic nerve ganglia are strategically located. Henderson et al. demonstrated how interventions like the rib rising technique can modulate ANS by altering salivary alpha-amylase levels, thus offering a valuable biomarker for the assessment of ANS functionality (2). The interdependence of these systems suggests that enhancing musculoskeletal parameters through osteopathic interventions can concurrently benefit pulmonary patients and those with nervous system disorders by improving neural regulation and muscle function.

Heart rate variability (HRV^4^) has emerged as the leading non-invasive measure for evaluating the response of ANS function to therapeutic interventions. It is remarkably patient-friendly, as it requires no active participation or exertion from the patient, making it an ideal tool for monitoring the long-term impact of therapies aimed at ameliorating ANS function (5–10). The use of HRV as a metric underscores the interconnected nature of osteopathic medicine, where improvements in autonomic regulation through manual techniques can lead to broader health benefits, including enhanced respiratory capacity and a more efficient musculoskeletal system. These broader health benefits are particularly relevant when considering osteopathic techniques such as cranial osteopathy, which have been the subject of much research in their potential to affect the cranial field and, by extension, the entire body. Cranial osteopathy, for instance, is a subtle yet impactful technique that has been scrutinized for its influence on the cranial field and, by proxy, its systemic effects on health and disease. The manipulation of the cranial field can have profound effects on the fluid dynamics within the central nervous system, potentially improving the flow of cerebrospinal fluid, which in turn could aid in the management of both neurological and musculoskeletal disorders. The most common cranial osteopathy technique is CV4^5^ (fourth ventricle compression), which involves a subtle, pulsating compression of the occipital region until achieving what is known as the Still Point, indicating a state of quietness.

The literature presents a variety of perspectives on the impact of osteopathic techniques, particularly in how they may affect the ANS, respiratory function, and disorders of the head-cervical-shoulder complex. Despite the prevalence of such treatments as HVLA manipulations, cranial field techniques, or rib lifting methods, there is no unanimity on their efficacy (5, 6, 11–13). This lack of consensus underscores the need for this review, which aims to integrate and scrutinize the extant research on the multifaceted effects of osteopathic techniques. By examining the impact these techniques have on ANS, respiratory function, and musculoskeletal disorders in a comprehensive manner, this review seeks to bridge the gaps in our understanding and offer a unified view of the benefits and limitations of osteopathic interventions.

Moreover, conducting a systematic review of these techniques within the realms of ANS and respiratory function is especially pertinent at this juncture, in light of the escalating interest in non-pharmacological interventions within healthcare settings. As we continue to unravel the intricacies of osteopathic medicine, it becomes progressively clear that judicious application of these techniques can significantly enhance a comprehensive approach to patient care. This not only encapsulates the direct effects on targeted tissues and systems but also embodies the impact on patient well-being, potentially acting as a valuable adjunct to pharmacological treatments and enhanced quality of life.

In this light, the current review is a pivotal endeavor to collate and evaluate the breadth of research concerning osteopathic manipulative treatments (OMTs). It will dissect the intricate relationships between the manipulation of bodily structures and the resultant changes in physiological parameters, offering insights into the mechanisms by which OMTs exert their effects. By incorporating a broad spectrum of studies, this review will provide a more nuanced understanding of how osteopathic techniques can be optimized for different conditions, ultimately contributing to a more tailored and effective treatment paradigm.

The objective of the study.

This review will therefore serve as a critical resource for both clinicians and researchers, guiding the integration of osteopathic techniques into medical practice and informing future investigations. It is the synthesis of such comprehensive research that will facilitate the advancement of osteopathy as a key component in the multidisciplinary approach to health and wellness.

There is no consensus in the literature as to whether the use of osteopathic techniques such as high velocity low amplitude (HVLA) manipulations performed within the cervical and thoracic spine, techniques aimed at the cranial field or the use of rib lifting techniques have a significant impact on ANS, respiratory function and head-cervical-shoulder complex disorders (5, 6, 11–15).

## 2 Survey methodology

### 2.1 Search strategy

For the purpose of the study, a review of studies assessing the impact of manual osteopathic techniques (OMT) on the state of the autonomic nervous system, spirometric parameters of the respiratory system and the state of the head-cervical-shoulder complex was performed. The following databases were used to search for works: PubMed, Virtual Health Library and Cochrane Central Register of Controlled Trials. The search strategy included keywords related to manual osteopathic treatment, autonomic nervous system, spirometry, respiratory function and head, neck and shoulder pain. An example of a search strategy for the PubMed database is shown in Table 1. All articles published between 2010 and 2023 were eligible for review.

**Table 1:** Search strategy (PubMed).

Search	Search terms
#1	“Osteopathic manual treatment” [Mesh]
#2	“Osteopathic therapy” [Title/Abstract] OR “osteopathic manual treatment” [Title/Abstract] OR “Osteopathy” [Title/Abstract] OR “Osteopathic medicine” [Title/Abstract]
#3	# 1 OR #2
#4	*“*Autonomic nervous system” [Text Word] OR “AUN” [Text Word]
#5	“Heart rate variability” [Text Word] OR “HRV” [Text Word] OR “heart rate” [Text Word] OR “HR” [Text Word]
#6	#4 OR #5

The sources of information were independently searched by two reviewers (JS and AK). Reviewers reviewed the identified articles and made inclusion decisions in accordance with the eligibility criteria. In the case of disputes, decisions were settled by consensus or through the involvement of a third reviewer (DC).

**Figure 1:**
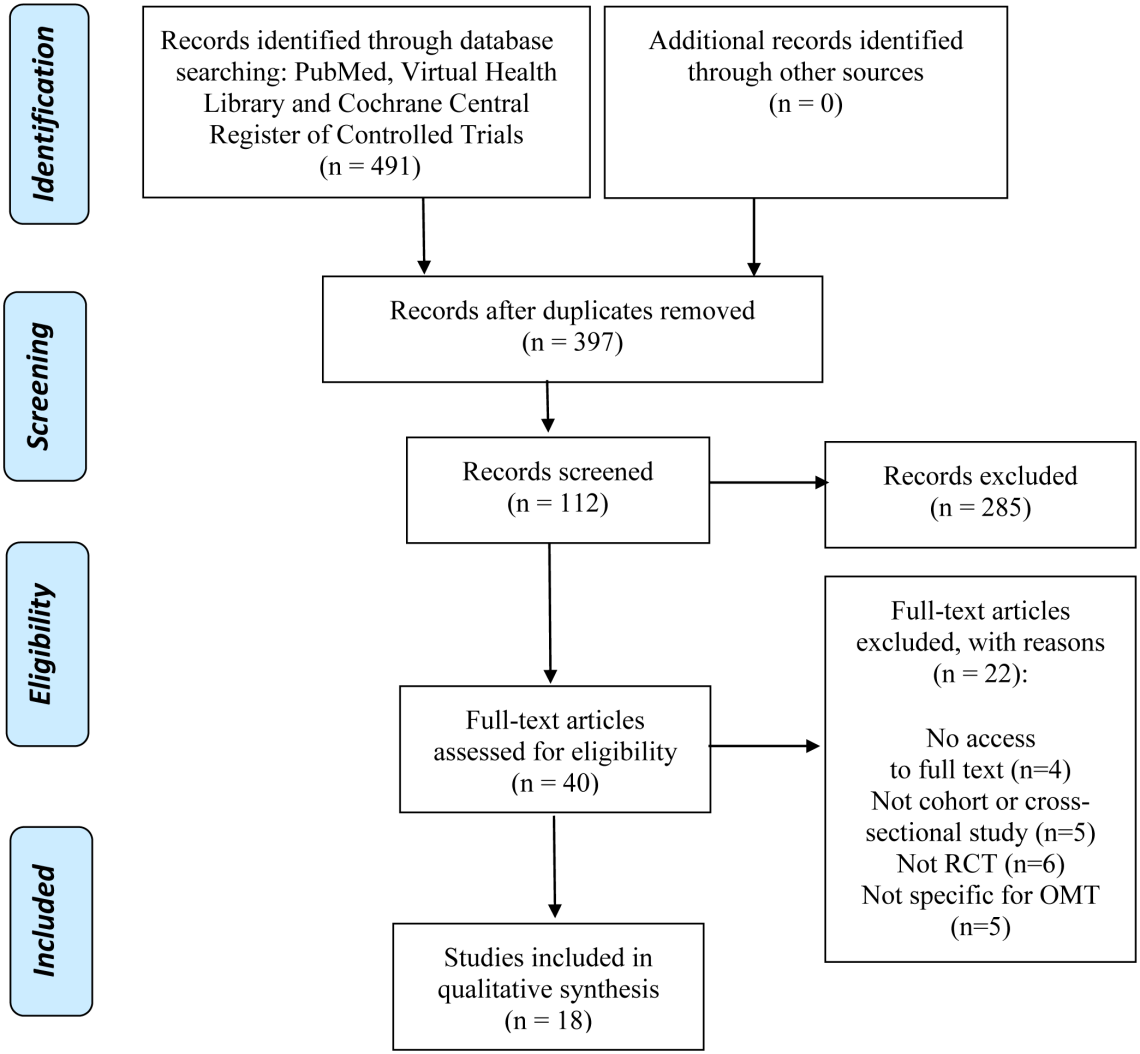
Flowchart of the included studies in this review.

All sources were analyzed in order to identify relevant studies, first based on the title, then based on the abstract and finally based on the full text. If the article could not be unequivocally excluded on the basis of title and abstract, after discussion between two independent reviewers, it was considered potentially relevant and included in the full text review (16). The number of articles included and excluded in each step is shown in the PRISMA flow diagram (Figure 1). The PRISMA guidelines (17, 18) were used for the systematic review.

### 2.2 Selection criteria

Works meeting the following criteria were included in the analysis:

- Randomized clinical trials, pilot studies;
- Experimental or intervention studies;
- Design of observational study (cohort or cross-sectional).

The following works were excluded from the study:

- Studies using case design and control;
- Review studies.
- Studies published in languages other than English;
- Monographs, post-conference materials.

The article has been registered in PROSPERO, the International prospective register of systematic reviews.

### 2.3 Data extraction

Data were obtained independently by two reviewers (JS and AK). Disputes were resolved through discussion between reviewers. The data included the first author, year of publication, study design, study population, participant characteristics, sample size, tools to measure autonomic nervous system, respiratory system and head-cervical and shoulder function. In the absence of potentially relevant data, the corresponding correspondent authors of the selected studies were contacted.

### 2.4 Quality assessment

The Downs and Black checklist for quality assessment of medical intervention studies was used to assess the articles included in this review (19). Each article was reviewed by two independent reviewers who used this scoring system. The Downs and Black result is designed to assess the quality of original research articles Tables 2–4.

**Table 2:** Summary of critical appraisal scores of the included studies (Downs and Black checklist).

	Study	Arsh et al. (2020) (11)	Masaracchio et al. (2013) (12)	Cholewicki et al. (2021) (13)	Stępnik and Czaprowski (2018) (20)	Corum et al. (2021) (21)	Groisman et al. (2020) (22)	Bautista-Aguirre et al. (2017) (23)
1	Reporting	Y	Y	Y	Y	Y	Y	Y
2		Y	Y	Y	Y	Y	Y	Y
3		Y	Y	Y	Y	Y	Y	Y
4		Y	Y	Y	Y	Y	Y	Y
5		N	Y	N	N	N	Y	N
6		Y	Y	Y	Y	Y	Y	Y
7		Y	Y	Y	Y	Y	Y	Y
8		N	N	N	N	N	N	N
9		Y	Y	Y	Y	Y	Y	Y
10		Y	Y	Y	Y	Y	N	Y
11	External validity	U	U	Y	Y	Y	Y	Y
12		U	U	Y	U	U	U	U
13		U	U	U	Y	U	U	Y
14		Y	Y	Y	Y	Y	Y	Y
15		Y	Y	Y	Y	Y	Y	Y
16		Y	Y	Y	Y	Y	Y	Y
17		Y	Y	Y	Y	Y	Y	Y
18		Y	Y	Y	Y	Y	Y	Y
19		Y	Y	Y	Y	Y	Y	Y
20		Y	Y	Y	Y	Y	Y	Y
21	Confounding	Y	U	U	Y	U	U	Y
22		Y	Y	Y	N	N	N	N
23		Y	Y	Y	Y	Y	Y	Y
24		Y	Y	Y	Y	Y	Y	Y
25		N	Y	N	N	N	Y	N
26		Y	Y	Y	Y	Y	Y	Y
27	Power	Y	Y	Y	Y	Y	Y	N
Total		21	22	22	22	20	21	21

Effect of OMT on head-cervical-shoulder complex ailments. Y, yes; N, no; U, unable to be determined.

**Table 3:** Summary of critical appraisal scores of the included studies (Downs and Black checklist).

	Study	Stępnik, Kędra and Czaprowski (2020) (14)	Swender et al. (2014) (15)	Jones et al. (2021) (24)	Abdelaal Ashraf et al. (2015) (16)	Buscemi et al. (2019) (25)
1	Reporting	Y	Y	Y	Y	Y
2		Y	Y	Y	Y	Y
3		Y	Y	Y	Y	Y
4		Y	Y	Y	Y	Y
5		N	N	N	N	Y
6		Y	Y	Y	Y	Y
7		Y	Y	Y	Y	N
8		N	N	N	N	Y
9		Y	Y	Y	Y	Y
10		Y	Y	Y	Y	Y
11	External validity	N	U	Y	U	Y
12		U	U	Y	Y	U
13		Y	Y	Y	Y	Y
14		Y	Y	Y	Y	Y
15		Y	U	U	Y	Y
16		Y	Y	Y	Y	Y
17		Y	Y	Y	Y	Y
18		Y	Y	Y	Y	Y
19		Y	Y	Y	Y	Y
20		Y	Y	Y	Y	Y
21	Confounding	U	Y	Y	Y	U
22		U	Y	Y	Y	Y
23		Y	Y	Y	Y	Y
24		U	U	U	U	U
25		N	N	N	N	Y
26		Y	Y	Y	Y	Y
27	Power	Y	N	Y	Y	Y
Total		19	19	22	22	23

Effect of OMT on respiratory system function. Y, yes; N, no; U, unable to be determined.

**Table 4:** Summary of critical appraisal scores of the included studies (Downs and Black checklist).

	Study	Cerritelli et al. (2020) (19)	Ruffini et al. (2015) (26)	Abenavoli et al. (2020) (18)	Arienti et al. (2010) (27)	Cardaso et al. (2014) (6)	Hendryx et al. (2022) (28)
1	Reporting	Y	Y	Y	Y	Y	Y
2		Y	Y	Y	Y	Y	Y
3		Y	Y	Y	Y	Y	Y
4		Y	Y	Y	Y	Y	Y
5		N	N	Y	Y	N	N
6		Y	Y	Y	Y	N	Y
7		Y	Y	Y	Y	Y	Y
8		N	N	N	N	N	N
9		Y	Y	Y	Y	Y	Y
10		Y	Y	Y	Y	Y	Y
11	External validity	N	U	N	U	Y	N
12		N	U	N	U	U	N
13		U	U	Y	U	U	U
14		Y	Y	Y	Y	Y	Y
15		U	U	Y	Y	Y	U
16		Y	Y	Y	Y	Y	Y
17		Y	Y	Y	Y	Y	Y
18		Y	Y	Y	Y	Y	Y
19		Y	Y	Y	Y	Y	Y
20		Y	Y	Y	Y	Y	Y
21	Confounding	Y	Y	Y	N	U	Y
22		Y	Y	U	Y	U	Y
23		Y	Y	Y	Y	Y	Y
24		N	N	Y	Y	Y	U
25		Y	Y	N	Y	Y	N
26		Y	Y	Y	Y	Y	Y
27	Power	Y	Y	Y	Y	Y	Y
Total		20	20	22	22	20	19

Effect of OMT on ANS functions. Y, yes; N, no; U, unable to be determined.

The checklist consists of 27 “yes” or “no” questions grouped into five sections: (1) the quality of the study (10 points), which assesses the overall quality of the study; (2) external validation (3 points) to determine whether the study results can be generalized; (3) the test error (7 points) to assess the error of the intervention and the results; (4) the impact of disrupters and selection (6 points) to determine the distortions resulting from the way the sample or group was selected; and (5) the power of the study (1 point) to determine whether the results are the result of chance (National Collaborating Center). Studies that received a result above 50% were taken for analysis.

## 3 Results

### 3.1 Search strategy

Four hundred and ninety-one potentially relevant articles were found as a result of a literature review. Of these, 228 were identified in PubMed, 84 in the Virtual Health Library, and 179 in Cochrane Central Register of Controlled Trials. After reviewing the abstracts, 40 potentially relevant articles were identified. At this stage, full text versions were obtained for further evaluation. After careful analysis, 23 articles that met the inclusion criteria were included in the qualitative synthesis, and 20 of them were qualified for meta-analysis. Finally, 18 works were included in the analysis, including:

- 15 randomized controlled trials,
- 3 pilot studies.

### 3.2 Study characteristic

#### 3.2.1 Effect of OMT on head-cervical-shoulder complex aliments

In seven randomized controlled trials investigating the effectiveness of OMT for addressing nonspecific cervical segment disorders or nonspecific headaches, the emphasis was mainly on functional disturbances. These disturbances encompassed myofascial dysfunctions and intervertebral joint disorders that impact the head-cervical-shoulder complex (12, 13, 20, 24, 26, 29, 30). The primary focus was on the effects of HVLA (high velocity, low amplitude) manipulative techniques on the cervical spine, mobilizations of the cervical spine’s facet joints, myofascial release techniques of the neck region, and stretching exercises. The total sample size was 461 people (from 31 to 97 participants). All studies recruited both women and men. The characteristics of the included studies are described in Table 5. Four of the included studies used the Numeric Pain Rating Scale (NPRS) (12, 13, 24, 30) and six used the Neck Disability Index (NDI) (12, 13, 20, 24, 27, 30) to assess the effectiveness of the procedures. In most studies, the authors showed statistically significant improvement in OMT groups (12, 13, 20, 24, 27, 30), while the Bautista-Aguirre et al. (29) study showed no significant difference between groups.

**Table 5:** Characteristics of the included studies.

	Design	Sample size	Intervention	Measurement	Main findings
**Effect of OMT on head-cervical-shoulder complex ailments**					
Arsh et al. (2020) (11)	Randomized controlled trial	37 subjects with nonspecific cervical spine pain 20- research group 17- control group	The control group received manual cervical and thoracic spine therapy for two weeks	Numeric pain rating scale (NPRS) and neck disability index (NDI)	There was a statistically significant difference between the groups in the reduction of pain (*p* = 0. 02) and disability (*p* = 0. 03)
Masaracchio et al. (2013) (12)	Pilot randomized controlled trial	64 subjects with mechanical pain in the cervical spine. The subjects were divided into two groups, in which two therapeutic sessions were performed each.	In the control group mobilizations of the cervical spine and a home program of stretching exercises were used, while in the experimental group manipulations of the thoracic spine were added.	Numeric pain rating scale (NPRS) and neck disability index (NDI)	Subjects from the experimental group achieved a statistically greater improvement (*p* = 0. 01) in the numerical scale of pain assessment and in the Neck Disability Index questionnaire
Cholewicki et al. (2021) (13)	Randomized controlled trial	97 people with chronic, nonspecific cervical spine pain. The subjects were divided into two groups, one receiving manual osteopathic therapy (OMT) and the other, a group waiting for therapy.	The OMT group received 3–4 therapeutic sessions over a period of 4–6 weeks. The study was completed by 38 subjects in the experimental group and 37 in the control group.	Numeric pain rating scale (NPRS), neck disability index (NDI), PROMIS-29 (Patient-Reported Outcomes Measurement Information System-29)	Subjects from the experimental group obtained a statistically significant improvement in the mean of experienced pain (−1.02, 95% confidence interval [CI] −1.72, −0.32; *p* = 0.005), current pain (−1.02, 95% CI −1.75, −0.30; *p* = 0.006), disability (−5.30, 95% CI −9.2, −1.3%; *p* = 0.010). There had also been improvements in the PROMIS-29 questionnaire parameters related to sleep(−3.25, 95% CI −6.95, −1.54; *p* = 0.003), fatigue (−3.26, 95% CI −6.04, −0.48; *p* = 0.022), and depression (−2.59, 95% CI −4.73, −0.45; *p* = 0.018)
Stępnik and Czaprowsk (2018) (20)	Randomized controlled trial	31 people with nonspecific cervical spine pain. Subjects were divided into two groups, experimental and control.	The experimental group received 5 osteopathic techniques (OMTs) for the anterior region of the neck, while the control group had a procedure performed on them with the laser switched off.	Neck disability index (NDI)	Statistically significant differences were shown in the experimental group by 8. 5 points (17%) (*p* > 0. 05)
Corum et al. (2021) (21)	Randomized controlled trial	54 people with tension-type headaches (TTH)	Each of the groups received eight treatment sessions: group 1-manipulation plus exercise (manipulation), group 2-suboccipital muscles inhibition plus exercise (facial release) and group 3-exercise only (control).	Headache frequency, pain severity (VAS headache, VAS neck pain) and disability associated with head and neck pain (HIT-6 and NDI respectively) were measured at baseline, after treatment and at month 3 of follow-up.	The manipulated group was statistically superior to the fascial release group in terms of headache frequency, headache severity and PPT (Pressure Pain Threshold) scores. Additionally, the manipulated group showed statistically significant improvements in all outcome criteria compared to the control group.
Groisman et al. (2020) (22)	Randomized controlled trial	90 adults with nonspecific chronic neck pain were randomly assigned to one of two groups: exercise (EG) or osteopathic manipulative therapy in combination with exercise (OMT/EG).	Each group received one exercise session per week with a physiotherapist for 4 weeks. The OMT group additionally received an osteopathic procedure lasting 50/60 min once a week.	Results were obtained using the Numeric Pain-Rating Scale (NPRS), Pressure Pain Threshold (PPT) and Neck Disability Index (NDI). Secondary outcomes included the range of motion (ROM) of the cervical spine rotation, the Fear-Avoidance Beliefs Questionnaire Work/Physical Activity (FABQ-W/PA) and self-efficacy in pain management at two different time points: at baseline and 4 weeks after the first treatment.	Patients in the osteopathic manipulative treatment group combined with exercise (OMT/EG) showed an increase in range of motion of neck rotation to both sides (ROM; *p* < 0. 05). This increase in range of motion did not occur in the exercise group (EG; *p* > 0. 05). Osteopathic manipulative treatment combined with exercise led to greater reductions in disability and pain compared to the exercise group. The OMT/EG group exhibited lower NPRS (mean difference, −1. 4; 95% CI -2. 4 to −0. 3; *p* = 0. 007), NDI (mean difference, −3. 8; 95% CI -0. 74 to −6. 9; *p* = 0. 01), and greater range of motion of neck rotation compared to patients in the exercise group (*p* < 0. 05)
Bautista-Aguirre et al. (2017) (23)	Randomized controlled trial	88 people with Grade I or II neck pain lasting at least 12 weeks	Participants were divided into three groups: (1) neck group; (2) chest group; and (3) control group. One treatment session was performed using the HVLA manipulation technique on the lower cervical spine (C7) or upper thoracic spine (T3), while the control group received an apparent manual intervention.	The pain-free grip strength was assessed with an isometric handheld hydraulic dynamometer. Mechanosensitivity was assessed using a digital dynamometer. Gradually increasing pressures of 1 kg/cm^2^/s were applied, with a 30-s interval between measurements, and the average of the three measurements was taken as a reference point. The pressure pain threshold was assessed on the nerves of the upper limb in the following order: (1) the medial nerve, at the wrist canal site and at the lower elbow; (2) ulnar nerve; (3) radial nerve.	In this randomized, controlled trial, overall results indicate that cervical or thoracic manipulation has a similar effect on upper limb mechanosensitivity and grip force in patients with chronic non-specific mechanical neck pain. However, all these changes were minor and below the threshold considered clinically relevant. Placebo showed similar results to spinal manipulation, which does not support the initial hypothesis.
**Effect of OMT on respiratory system function**					
Stępnik and Czaprowski (2020) (14)	Randomized controlled trial	30 healthy people were divided into two groups - experimental and placebo	The participants from the experimental group were treated with such osteopathic techniques as the thoracic thrust (manipulations of vertebral joints and ribs), the sternal pump technique and stretching of the diaphragm. The placebo group was treated with soft tissue therapy (STT) techniques for the masseter muscle	Spirometry	The experimental group showed a significant change in PEF (Peak Expiratory Flow) parameter (*p* = 0. 0001) but did not affect FVC (Forced Vital Capacity) and FEV1 (Forced Expiratory Volume in 1 s) parameters.
Swender et al. (2014) (15)	Single-blind randomized controlled trial	33 subjects diagnosed with cystic fibrosis divided into two experimental groups (OMT therapy + standard treatment) and control (standard treatment)	Manipulation protocol included: rib elevation, abdominal diaphragm release, thoracic inlet muscle release, thoracic lymph pump and suboccipital decompression.	Spirometry and questionnaire data (self-assessment of breathing, pain, and anxiety level)	There were no differences between the OMT group and the FEV1% simulated group. There was a significant change between the two groups in the questionnaire on breathing, pain and anxiety. Fifteen out of 16 in the OMT group showed improvement, while only 8 out of 16 in the sham group reported a positive change	
Jones et al. (2021) (24)	Clinical trial	58 children aged 7–18 diagnosed with asthma, were divided into an experimental group, where in addition to standard care they received osteopathic manual therapy and a control group that received only standard care	The handling protocol included: rib lift and suboccipital release	Spirometry	Subjects showed improvement in FEV1% and FEV, although this was not statistically significant. The PEF parameter was not tested.	
Abdelaal Ashraf et al. (2015) (16)	Randomized controlled trial	195 male COPD patients were randomized to the diaphragm manipulation group (Group A; *n* = 46), rib lifting group (Group B; *n* = 53), both procedures group (Group C; *n* = 50), and control group (Group -D; *n* = 46).	12-week protocol, comprising two procedures per week to examine ventilation (VF) and functional capacity (FC) responses to diaphragm or rib manipulations or both	The parameters assessed included FVC in liters, FEV1 in liters and patient walking distance for 6 min (6MWT) in meters.	At the end of the study, mean FVC, FEV1 and 6MWT values and percent increases were [3. 63 ± 0. 56 (4. 52%), 2. 46 ± 0. 51 (14. 42%), 416. 35 ± 28. 62 (3. 82%)] for group A, [3. 56 ± 0. 38 (5. 97%), 2. 43 ± 0. 48 (16. 63%), 415. 28 ± 37. 81 (3. 04%)] for group B and [3. 93 ± 0. 54 (16. 92%), 2. 86 ± 0. 5 (33. 44%), 433. 03 ± 46. 76 (6. 9%)] for group C (*p* < 0. 05). There were also significant differences in mean FVC, FEV1 and 6MWT values between the groups at the end of the study, but in favor of Group C (*p* < 0. 05).	
Buscemi et al. (2019) (25)	Randomized controlled pilot study	32 patients with moderate to severe chronic obstructive pulmonary disease (COPD). The control group received standard pharmacological treatment and the study group received pharmacological treatment and osteopathic therapy OMT	4 OMT sessions, the OMT protocol included muscle-fascial release techniques for the treatment of maxillary sinuses, vertebra-pulmonary ligaments, diaphragm nerves, ribs, pleural, lung, bronchial, subclavian muscles and cone-circular ligaments to improve the function of the chest and its adjacent structures	Spirometry, COPD Assessment Test (CAT) and six minutes walking test (6MWT)	Subjects from the OMT group received better results in all trials. FVC (*p* < 0.5411), entire FEV1 (*p* < 0.5061); CAT: OMT (*p* < 0.0005) - control group (*p* < 0.188) 6MWT OMT (*p* < 0.0038) - control group (*p* < 0.5326)	
Effect of OMT on ANS function						
Cerritelli et al. (2020) (19)		Randomized controlled trial	37 healthy people. Subjects were divided into two groups, experimental and simulated group. Experimental group had been given therapy once a week for two weeks.	In the experimental group the techniques performed were not standardized, the therapist looked for tissue changes, asymmetry, range of motion and tenderness parameters. The therapist looked for disorders throughout the body and treated them one by one. In the simulated group, the therapist placed his hand on the patient’s body, applying gentle static pressure on different areas of the body.	Subjects were subjected to thermal imaging of the face, dermal conductivity and HRV before and after the trial.	The subjects in the OMT group showed a statistically significant improvement in the temperature increase of individual facial regions (excluding the cheek region), improvement in the parameters of dermal conductivity and improvement in the parameter of HF (high frequencies – corresponding to the activity of the parasympathetic system). In the simulated group, there was no improvement in any of the parameters
Ruffini et al. (2015) (26)		Randomized controlled trial	66 healthy people were divided into three groups. The first group received OMT therapy in the first session and simulated therapy in the second, the second group received simulated therapy in the first session and OMT in the second, while the third group received no therapy.	The OMT protocol was not standardized and it was based on patient needs.	HRV study, parameters HF and LF	The results showed a statistically significant improvement for groups 1 and 2 after OMT in the increase in HF (related to parasympathetic system activity) and decrease in LF (related to sympathetic system activity). In groups 1 and 2 on simulated therapy, and in group 3, the groups showed no difference in parameters.
Abenavoli et al. (2020) (18)		A pilot randomized single blind clinical trial	90 subjects were randomized to placebo, control or CV4 group	The experimental group had the CV4 technique performed, the Sham CV-4 group had the simulated CV4 technique performed and the control group sat still for 15 min.	Participants were seated and rinsed their mouths with clean water. They collected saliva and emptied their mouths by salivating into a 15-milliliter polypropylene tube for five minutes. Saliva was collected before (t0), immediately after (t1) and 30 min after (t2) CV4 or placebo/control procedures.	Salivary alpha amylase activity increased between t0 and t2 in all groups, but only in CV4 subjects the increase immediately after the technique was statistically significant.
Arienti et al. (2010) (27)		Randomized controlled trial	32 healthy volunteers divided into three groups	Participants were divided by a simple draw and assigned by the blinded investigator to three groups: group CV4 (CV4) - 16 subjects submitted to the CV4 technique; group Rib Raising (RR) - 10 subjects submitted to the Rib Raising technique; group Placebo (PL) - 6 subjects submitted to the mild touch of placebo.	HRV and dermal conduction	Results showed an increase in HF power and LF/HF ratio, suggesting a shift in autonomic balance toward a dominant parasympathetic state, as indicated by changes in heart rate variability (HRV) during CV4 compared to placebo in healthy subjects.
Cardaso et al. (2014) (6)		Randomized controlled trial	40 healthy volunteers divided into two groups	Forty healthy adults were randomly assigned to the CV-4 intervention group or to the placebo (non-therapeutic occipital bone contact) control group. The duration of each procedure was 10 min	In both groups, plasma catecholamine levels, blood pressure and heart rate were measured before and immediately after the intervention.	No intervention-related effects were observed. Although minimal decreases in noradrenaline, systolic blood pressure and heart rate were observed after the intervention, this was not exclusively related to the intervention group. In fact, only the control group showed a statistically significant increase in dopamine levels after the intervention.
Hendryx et al. (2022) (28)		Randomized controlled trial	80 healthy volunteers, out of which 77 completed the study.	Forty healthy adults were randomly assigned to the CV-4 intervention group or to the placebo (non-therapeutic occipital bone contact) control group.	Using AMI (Apparatus for Meridian Identification), bioelectrical skin parameters of specific acupuncture points on hands and feet were measured immediately before and after CV4 or placebo treatments.	After CV4 treatment, AMI measurements indicating the average value of left and right side measurements were reduced at 13 of 14 Ting acupuncture points, and this difference was statistically significant (*p* < 0. 05). After the placebo procedure, AP measurements were decreased at five Ting acupuncture points, increased at two points and unchanged at seven points; none of these differences were statistically significant (*p* ≥ 0. 05).	

#### 3.2.2 Effect of OMT on respiratory system

In five RCT studies on the effects of OMT on the respiratory system (11, 15, 21, 22, 28). The total sample size was 348 people (from 30 to 195 participants). The primarily studied techniques were: HVLA manipulations of the thoracic spine and costotransverse joints, rib elevation, abdominal diaphragm release, thoracic inlet muscle release, thoracic lymph pump, and suboccipital decompression. All studies recruited both women and men. The characteristics of the included studies are described in Table 5.

Two studies were performed on people with Chronic Obstructive Pulmonary Disease (COPD) (11, 28), one on children with bronchial asthma (21), one with cystic fibrosis (22) and one on healthy people (15). In all included studies (*n* = 5), spirometry (11, 15, 21, 22, 28) was used to assess the efficacy of the procedures used. One study additionally used a questionnaire (Self-Assessment of Breathing, Pain, and Anxiety Level) (22) and two 6 min Walking test (6MWT) (11, 28). All studies (11, 15, 21, 28) except one (22) showed a statistically significant difference between OMT and control groups.

#### 3.2.3 Effect of OMT on ANS function

In six RCT studies on the effects of OMT on autonomic nervous system function (9, 10, 23, 25, 26). In most studies, the impact of the CV4 technique and the Respiratory Rib (RR) technique was examined, while in some studies, techniques were tailored to the needs of the subject. The total sample size was 365 people (from 32 to 90 participants). All studies recruited both women and men. The characteristics of the included studies are described in Table 5. Three RCT studies (9, 10, 26) used HRV to evaluate the efficacy of the procedures, two (9, 26) additionally measured the level of dermal conductivity, one (23) tested the level of salivary alpha amylase, one tested the level of plasma catecholamines and one (25) measured the bioelectrical parameters of the skin of specific acupuncture points on the hands and feet.

Most studies showed statistically significant improvement in OMT groups (9, 10, 23, 25, 26), while Cardoso et al. (5) did not show a significant difference between groups.

In systematic review studies Roura et al. (31) found that manual therapy may affect both the sympathetic and parasympathetic systems, but the results were inconsistent, the methodology was heterogeneous, and there were significant differences in how the effects were measured. They concluded that the impact of manual therapy on the autonomic nervous system remains unclear and offered their guidance for future studies. Żurowska et al. (16) found on the basis of the analyzed studies a significant impact of CV4 technique on various physiological parameters and autonomic system function (ANS), cerebral cortex activity (especially in the alpha band), pain assessment (visual analog scale) and falling asleep faster.

## 4 Discussion

Osteopaths use osteopathic manipulative therapy (OMT) to treat somatic dysfunctions such as tissue lesions, range of motion limitations and asymmetry (16). It is claimed that OMT induces anti-inflammatory effects and increases the activity of the parasympathetic system (16). OMT practitioners use a variety of techniques and manual approaches, including joint, fascial, visceral and cranial osteopathy (16). The selection and criteria helped to identify 20 scientific publications that presented high quality research. A systematic review showed the effectiveness of OMT therapy on the autonomic nervous system, respiratory system function and the condition of the head-cervical-shoulder complex (9, 11–13, 15, 20, 21, 24–28). In the case of the study of the effect of OMT therapy on the condition of the head-cervical-shoulder complex and the function of the respiratory system, the studies seem to be more conclusive showing the effectiveness of the therapy (11–13, 15, 20–22, 24, 27, 28, 30). This may be due to easier selection of techniques and standardization of therapeutic protocols. ANS is a much more complex system that affects the whole body and all body systems, so it may be more difficult to choose a single technique or a generalized therapeutic protocol. Cerritelli et al. (26) and Ruffini and et al. (10) in their studies did not use standardized therapeutic protocols, but rather individually tailored therapy to the needs of each patient. In terms of the results, it turned out to be the most effective and the most similar to the therapy performed by osteopaths on a daily basis. The downside of these studies is that we do not know exactly what worked on the subjects and therefore it is difficult to verify what worked and what did not. The most frequently studied OMT technique to improve ANS status is CV4 (9, 16, 23, 25). It is possible that this is the reason for the ambiguity in the results of the study, because through it we mainly influence the ANS and the vagus nerve centrally. Cerritelli et al. (26) and Ruffini et al. (10) have shown that whole-body therapy may have a better effect on ANS function than single-region therapy. The weakness of studies on the impact of OMT on ANS function is the number of interventions. Most often the effect is studied after one treatment (5, 10, 23, 25) or two treatments (26). This may be too small a number of interventions to achieve a significant and lasting therapeutic effect. The most common study to assess ANS status was HRV (9, 10, 26). Heart Rate Variability (HRV) is an indicator of the autonomic nervous system’s role in heart regulation, reflecting the heart’s ability to adapt to various physiological and environmental situations. HRV refers to the variations in the intervals between consecutive heartbeats (known as RR intervals), visible on an ECG recording. High HRV indicates a healthy cardiovascular system with a strong ability to adapt, whereas low HRV may suggest excessive nervous system burden, stress, or other health issues (32).

Main HRV Parameters (32):

Power spectral density (PSD) components:

- ULF (Ultra Low Frequency): Component below 0.003 Hz, potentially related to factors like thermoregulation mechanisms and slow metabolic processes.
- VLF (Very Low Frequency): Component from 0.003 to 0.04 Hz, whose precise significance is not fully understood but may include thermoregulatory processes, hormonal effects, and responses to inflammatory processes.
- LF (Low Frequency): Component from 0.04 to 0.15 Hz, considered an indicator of sympathetic activity, though it is now recognized to reflect both sympathetic and parasympathetic activity. Low HF levels can be correlated with anxiety, stress, panic and worry (5).
- HF (High Frequency): Component from 0.15 to 0.4 Hz, associated with respiration and considered a marker of parasympathetic activity.

Time domain methods:

- SDNN (Standard Deviation of NN intervals): The standard deviation of all NN intervals (normal to normal), reflecting overall HRV activity.
- RMSSD (Root Mean Square of the Successive Differences): The root mean square of successive differences between NN intervals, indicating parasympathetic activity.

Geometric methods: Analyze the geometric structure of the tachogram, such as the HRV triangular index, which relates to the total number of NN intervals divided by the maximum number of NN intervals in the modal bin of the histogram.

The downside of the HRV test is the instability of the results depending on the subject’s current psychological state, which is dependent on current life experiences and stress, over which the researchers have no influence.

Currently, there is no clear agreement as to whether osteopathic manual techniques (OMT) can significantly affect ANS function, although most scientific studies show their beneficial effect (9, 10, 23, 25, 26). Studies show the effect of OMT on the ranges of motility of the head-cervical-shoulder complex and spirometric parameters (11–13, 15, 22–28).

OMT applied to the head–neck-shoulder complex, as well as to the respiratory system and the autonomic nervous system (ANS), may exert influence through a variety of physiological and neurological pathways. Here are some mechanistic hypotheses that might explain the observed effects:

- Improvement of ANS Function: Manual techniques such as the compression of the fourth ventricle (CV4) and rib raising may modulate the balance between sympathetic and parasympathetic activity of the ANS. The mechanistic hypothesis suggests that by reducing tissue tension and normalizing structural positions, OMT may decrease physiological stress and affect body homeostasis. The reduction in tension may in turn lead to a decrease in sympathetic activity and an increase in parasympathetic activity, leading to improved heart rate variability (HRV) and overall nervous system reactivity.
- Improvement of Respiratory System Function: Techniques such as thoracic spine manipulation, diaphragm mobilization, and lymphatic techniques may improve spirometric parameters by increasing chest mobility, reducing airway resistance, and optimizing gas exchange. A possible mechanism is the improvement of breathing biomechanics through the reduction of respiratory muscle tension and improved diaphragm mobility, which translates into more efficient lung ventilation.
- Treatment of Dysfunctions in the Head–Neck-Shoulder Complex: OMT, including HVLA manipulations, soft tissue techniques, and mobilizations, may provide pain relief and improved range of motion through mechanisms such as reducing muscle tension, improving local circulation, and reducing inflammation. The mechanistic hypothesis assumes that by normalizing anatomical and functional relationships within this complex, OMT can affect the reduction of nociceptive activity and modulation of pain.

## 5 Conclusion

Despite the impact of OMT on both spirometric parameters and the condition of the head-collar-shoulder complex, this does not always translate into improved ANS performance. Most scientific studies show a beneficial effect of OMT therapy on the physiological parameters of patients, but there is no unequivocal agreement on this claim. Due to the complexity of ANS, it is possible that using a unified treatment protocol for all subjects may not be the best solution and it would be worth choosing the treatment protocol individually for each subject, as did Cerritelli et al. (26) and Ruffini et al. (10).

When considering the mechanisms of action of OMT, it is important to emphasize the interdisciplinary nature of these interventions, which combine neuromusculoskeletal, somatovisceral, and psychoneuroimmunological elements. Further research, including experimental studies, is necessary to fully understand the mechanisms through which OMT exerts its therapeutic effect. By integrating clinical data with advanced research techniques, such as functional imaging, we can better understand and harness the potential of osteopathic manual techniques in promoting health and treating diseases.

## Data availability statement

The original contributions presented in the study are included in the article/Supplementary material, further inquiries can be directed to the corresponding author.

## Author contributions

JS: Conceptualization, Data curation, Formal analysis, Investigation, Methodology, Project administration, Visualization, Writing – original draft, Writing – review & editing. DC: Conceptualization, Formal analysis, Methodology, Supervision, Writing – original draft, Writing – review & editing. AK: Conceptualization, Data curation, Formal analysis, Investigation, Methodology, Writing – original draft, Writing – review & editing.

## Footnotes

^1^AUN–autonomic nervous system.

^2^Head-cervical-shoulder complex–This concept encompasses a complex of tissues including the occipital bone, cervical vertebrae, scalene muscles, sternocleidomastoid muscle, trapezius, deep neck muscles, brachial plexus, scapula, shoulder joint, and the muscles surrounding the shoulder joint.

^3^HVLA–high-velocity low-amplitude manipulations is a manual therapy technique commonly used in osteopathy and chiropractic care. It involves a quick and precise thrust applied to a restricted joint to restore its normal range of motion, often accompanied by a popping sound due to the release of gases from the joint.

^4^HRV–Heart rate variability.

^5^CV4–fourth ventricle compression. The CV4 technique in osteopathy involves a gentle, rhythmic compression applied to the occipital region to induce a state of stillness known as the Still Point. This method aims to promote relaxation and restore balance within the cranial system.
